# Molecular evidence of *Borrelia theileri* and closely related *Borrelia* spp. in hard ticks infesting domestic animals

**DOI:** 10.3389/fvets.2023.1297928

**Published:** 2023-11-28

**Authors:** Mehran Khan, Mashal M. Almutairi, Abdulaziz Alouffi, Tetsuya Tanaka, Shun-Chung Chang, Chien-Chin Chen, Abid Ali

**Affiliations:** ^1^Department of Zoology, Abdul Wali Khan University Mardan, Khyber Pakhtunkhwa, Pakistan; ^2^Department of Pharmacology and Toxicology, College of Pharmacy, King Saud University, Riyadh, Saudi Arabia; ^3^King Abdulaziz City for Science and Technology, Riyadh, Saudi Arabia; ^4^Laboratory of Infectious Diseases, Joint Faculty of Veterinary Medicine, Kagoshima University, Kagoshima, Japan; ^5^Department of Emergency Medicine, Ditmanson Medical Foundation Chia-Yi Christian Hospital, Chiayi, Taiwan; ^6^Department of Pathology, Ditmanson Medical Foundation Chia-Yi Christian Hospital, Chiayi, Taiwan; ^7^Department of Cosmetic Science, Chia Nan University of Pharmacy and Science, Tainan, Taiwan; ^8^Ph.D. Program in Translational Medicine, Rong Hsing Research Center for Translational Medicine, National Chung Hsing University, Taichung, Taiwan; ^9^Department of Biotechnology and Bioindustry Sciences, College of Bioscience and Biotechnology, National Cheng Kung University, Tainan, Taiwan

**Keywords:** hard ticks, *Borrelia*, *Borrelia theileri*, domestic animals, Pakistan

## Abstract

Ticks pose significant threats to hosts by transmitting *Borrelia* spp., which are grouped into Lyme borreliae, relapsing fever borreliae (RF), and reptiles- and monotremes-associated borreliae. The RF borreliae encompass a group of *Borrelia* species predominantly transmitted by soft ticks, but some of its members can also be transmitted by hard ticks. Information on the detection and genetic characterization of tick-borne RF borreliae, including *Borrelia theileri*, is notably rare in Asia, particularly in Pakistan. Herein, we employed molecular techniques to detect borreliae in hard ticks collected from domestic animals in Khyber Pakhtunkhwa, Pakistan. Ticks were subjected to morphological analysis, followed by DNA extraction and PCR amplification of partial fragments of borrelial 16S rRNA and *flaB* genes. A total of 729 ticks were collected from 264 hosts, with *Haemaphysalis cornupunctata* (12.9%; 94/729) being the most prevalent, followed by *Hyalomma anatolicum* (11.7%; 85/729), *Rhipicephalus microplus* (10.0%; 73/729), *Haemaphysalis kashmirensis* (9.1%; 66/729), *Haemaphysalis bispinosa* (8.5%; 62/729), *Rhipicephalus sanguineus* (8%; 58/729), *Haemaphysalis montgomeryi* (6.2%; 45/729), *Rhipicephalus turanicus* (5.5%; 40/729), *Hyalomma dromedarii* and *Ixodes kashmirensis* (4.4%; 32/729 each), *Rhipicephalus haemaphysaloides* (4.1%; 30/729), *Haemaphysalis sulcata* and *Hyalomma scupense* (3.8%; 28/729 each), *Haemaphysalis danieli* (2.9%; 21/729), *Hyalomma kumari* (2.6%; 19/729), and *Hyalomma isaaci* (2.2%; 16/729). Based on 16S rRNA detection of *Borrelia* spp., only *R. turanicus* yielded positive results, resulting in an overall infection rate of 0.3% (2/160), while using *flaB*-based detection, four tick species including *R. microplus*, *R. turanicus*, *Ha. sulcata*, and *Ha. cornupunctata* showed positive results, yielding an overall infection rate of 6.9% (11/160). The amplified DNA fragments of borrelial 16S rRNA and *flaB* in *R. turanicus* from goats shared maximum identities of 100 and 99.40% with *Borrelia theileri*, respectively. Amplified borrelial *flaB* fragments in *R. microplus* from cows and sheep displayed 100% identity with *B. theileri*, while *flaB* fragments in *Ha. cornupunctata* and *Ha. sulcata* from goats revealed identities of 99.32 and 99.75% with undetermined RF *Borrelia* spp., respectively. Phylogenetic analysis revealed clustering of *B. theileri* from *R. microplus* and *R. turanicus* with the same species, while *Borrelia* spp. from *Ha. cornupunctata* and *Ha. sulcata* with undetermined RF *Borrelia* spp. Notably, this research marks the first documentation of *B. theileri* in *R. turanicus* and the identification of RF *Borrelia* spp. in *Ha. cornupunctata* and *Ha. sulcata*.

## Introduction

Ticks are voracious blood feeders of all classes of terrestrial vertebrates (1, 2). They are distributed worldwide and they typically thrive more in warm and humid climates (1–4). Through various means, ticks can harm their vertebrate hosts, including the transmission of pathogens, such as *Borrelia* spp. (5–7).

*Borrelia* is a diverse genus of Gram-negative bacteria that act as obligatory parasites (8–10). *Borrelia* spp., alternate between arthropod vectors, including ticks, and vertebrate hosts such as domestic animals (8, 10). Due to their pathogenicity to vertebrate hosts, including humans, some *Borrelia* spp., are considered of significant global health concern, as they cause emerging and reemerging infectious diseases (11, 12). With approximately 42 known species, *Borrelia* spp., are divided into three main categories: Lyme borreliae (LB), relapsing fever borreliae (RF), and reptile-associated (REP) and monotreme associated borreliae (MON) (10, 11, 13–20). Owing to the diversity of spirochetes in the genus *Borrelia*, there have been proposals to split it into different genera (21, 22), however, this remains a controversial debate (10, 23).

Relapsing fever borreliae are endemic in temperate and tropical regions of the world, including Asia (8, 24). These agents are typically transmitted by soft ticks belonging to the genus *Ornithodoros* (25, 26). However, some members of RF borreliae including *Borrelia theileri*, *Borrelia miyamotoi* and *Borrelia lonestari* are primarily transmitted by hard ticks (26–29). With approximately 21 known species, RF borreliae are usually categorized into three groups: soft tick relapsing fever (STRF), hard ticks relapsing fever (HTRF), and louse-borne relapsing fever borreliae (6, 26). Relapsing fever borreliae are maintained in enzootic cycles covering birds and mammals (30). With the exception to *Borrelia duttonii*, which is originally associated with humans, other RF borreliae accidently infect humans.

Among the TBRF borreliae, *B. theileri* is the causative agent of bovine borreliosis in livestock including cows, goats and sheep (6, 31–34). This bacterium is worldwide distributed and transmitted by various hard tick species, mainly belonging to the genus *Rhipicephalus* (6, 26, 35).

The pathogenicity of *B. theileri* in domestic animals is known (36, 37), while there is a notable diversity and abundance of their vertebrate hosts and tick vectors in Pakistan (38–42). Concern exists regarding the negative impacts of this pathogen on the country’s livestock industry, though this impact has yet to be determined. In continuation with our previous studies, which detected REP *Borrelia* sp. and *Borrelia anserina* (43, 44) in ticks, the objective of this study was to genetically characterize RF borreliae in hard ticks infesting domestic animals.

## Materials and methods

### Study area

**Figure 1:**
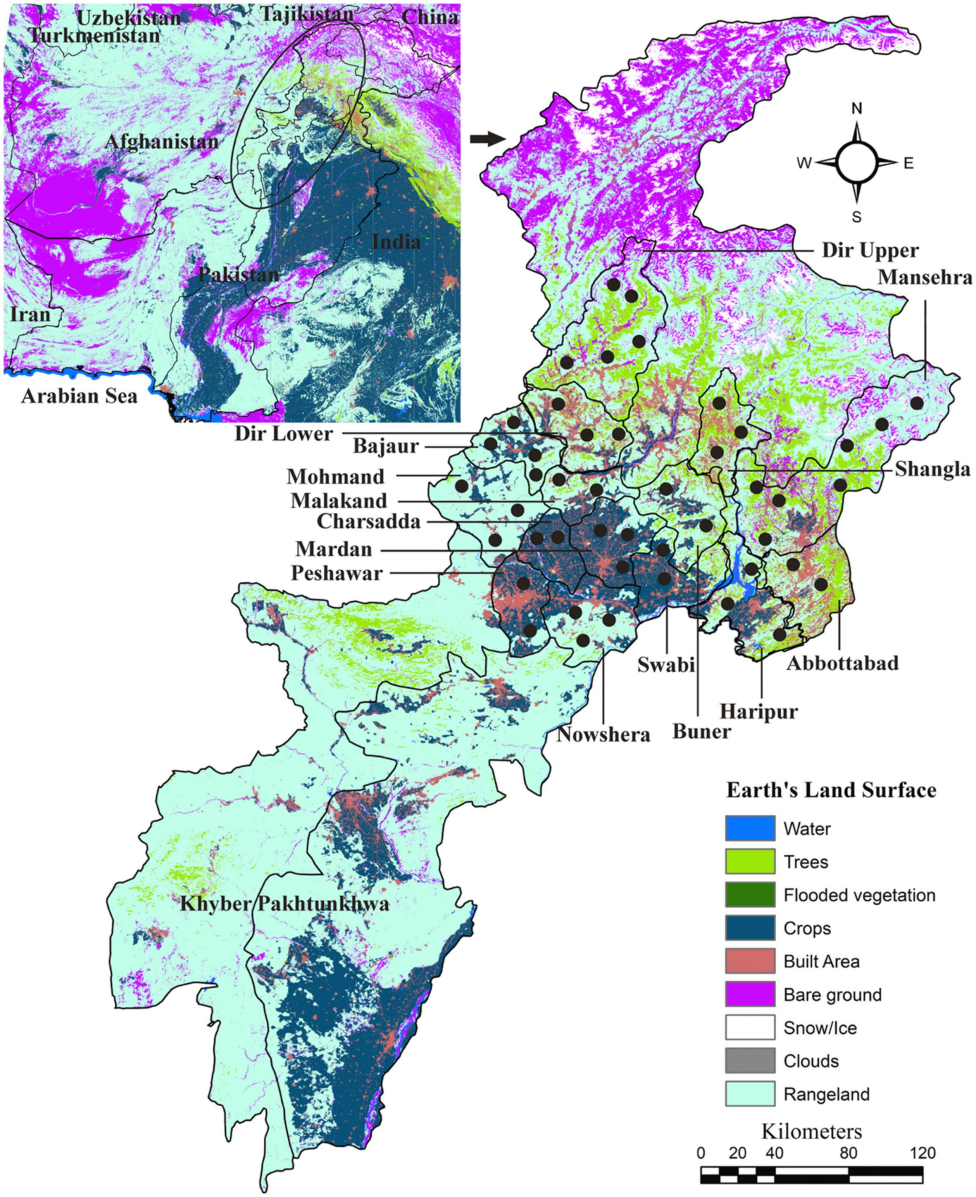
Land-use and land-cover based map showing locations (black circles) where domestic animals were screened for collecting ticks.

*In toto*, 15 districts of Khyber Pakhtunkhwa, including Abbottabad, Bajaur, Buner, Charsadda, Dir Lower, Dir Upper, Haripur, Malakand, Mardan, Mohmand, Nowshera, Peshawar, Shangla, Swabi, and Swat, were included in this study. Google Maps was utilized to determine the precise geographical coordinates of the collection points in the study area, and these information were organized in Microsoft Excel 2016. The land-cover map of the study area was created using ArcGIS version 10.3.1 (ESRI, Redlands, CA, United States; Figure 1).

### Tick collection, preservation, and identification

The study was conducted from March to September 2022, covering three seasons in Pakistan: spring (March–May), summer (June–August), and early autumn (September). Different domestic animals were screened for ticks in farms and grazing fields, when found, ticks were collected using tweezers. Essential information, including the host type, collection date, and collection site coordinates, was recorded during fieldwork. Before preservation in 70% ethanol, all collected ticks were washed with distilled water followed by 70% ethanol. Tick specimens were morphologically identified up to the species level using a stereomicroscope (Stemi 508, Zeiss, Germany) and standard taxonomic identification keys and morphological descriptions (45–51).

### DNA extraction and polymerase chain reaction

A subset of 160 specimens, consisting of at least 1 female per tick species per district and 3 nymphs per tick species, underwent DNA extraction. Tick homogenization was performed individually using sterile scissors in 1.5 mL Eppendorf tubes. DNA extraction was carried out using the phenol-chloroform method with minor modifications (52). The extracted DNA was then hydrated by adding 20–30 μL of “nuclease-free” water, and its quantification was analyzed using a NanoDrop spectrophotometer (Nano-Q, Optizen, South Korea).

All extracted genomic DNA samples were subjected to a conventional PCR (BIOER, China). Partial fragments of borrelial 16S rRNA were amplified through standard PCR, while borrelial *flaB* amplification was achieved through nested-PCR. For 16S rRNA and the first round of *flaB* PCR, the reaction mixture had a total volume of 25 μL and included components: 2 μL of extracted DNA (50–100 ng/μL), 1 μL of each forward and reverse primer (Table 1) at a concentration of 10 pmol/μL, 8.5 μL of nuclease-free PCR water, and 12.5 μL of Dream*Taq* green MasterMix (2X). The second round of *flaB* PCR was carried out with 1 μL of the first round PCR product and 9.5 μL of PCR water. All PCRs were conducted under experimental conditions, as previously described (43). PCR water (nuclease-free) was used as a negative control, while DNA of *Borrelia* sp. from *A. gervaisi* ticks as a positive control. The amplified fragments were resolved on a 2% agarose gel, visualized using a Gel Documentation system (BioDoc-It^™^ Imaging Systems, Upland, CA, United States), and purified using the GeneClean II Kit (Qbiogene, Illkirch, France).

**Table 1:** Primers used for amplification of targeted DNA of *Borrelia* spp. by conventional PCR.

Gene	Primer	Primer sequence (5′ to 3′)	Amplicons size (bp)	Reference
16S rRNA	S5-F	GAGGAATAAGCTTTGTAGGA	746	Fleche et al. (53)
	S13-R	ACGTCATCCTCACCTTCCT		
*flaB*	Fla LL	ACATATTCAGATGCAGACAGAGGT	665	Stromdahl et al. (54)
	Fla RL	GCAATCATAGCCATTGCAGATTGT		
	Fla LS*	AACAGCTGAAGAGCTTGGAATG	354	Stromdahl et al. (54)
	Fla RS*	CTTTGATCACTTATCATTCTAATAGC		

*Used in a second round/nested reaction.

### DNA sequencing and phylogenetic analysis

The amplicons were sent for DNA sequencing (Macrogen, Inc., Seoul, South Korea) using the Sanger sequencing method with an ABI 373XL system. The raw sequences obtained were then visualized and analyzed using SeqMan version 5.0 (DNASTAR, Inc., Madison, WI, United States) to obtain clean sequences. These clean sequences were subsequently subjected to BLAST (Basic Local Alignment Search Tool) analysis at the NCBI (National Center for Biotechnology Information) to identify the closest matches with sequences already deposited in the GenBank.

The obtained sequences, homologous sequences (downloaded from the BLAST results), and an appropriate outgroup were imported and aligned using the BioEdit alignment editor version 7.0.5 (55) with the ClustalW multiple alignment method (56). The tree topology was adapted from previous studies (10, 13, 16, 20, 43). The alignments were then used in Molecular Evolutionary Genetics Analysis (MEGA-X) (57) to construct phylogenetic trees, utilizing the Maximum Likelihood method with 1,000 bootstrap replicates.

## Results

### Tick and host description

Of the total collected specimens (729), *Haemaphysalis* ticks were the most abundant comprising 43.3% (316/729), followed by the *Rhipicephalus* ticks (27.6%, 201/729), *Hyalomma* (24.7%, 180/729) and *Ixodes* ticks (4.4%, 32/729). Within the genus *Haemaphysalis*, the most abundant species was *Haemaphysalis cornupunctata* (29.7%; 94/316) followed by *Haemaphysalis kashmirensis* (20.9%; 66/316), *Haemaphysalis bispinosa* (19.6%; 62/316), *Haemaphysalis montgomeryi* (14.2%; 45/316), *Haemaphysalis sulcata* (8.9%; 28/316), and *Haemaphysalis danieli* (6.6%; 21/316). Among *Rhipicephalus* ticks, the most abundant species was *Rhipicephalus microplus* (36.3%; 73/201) followed by *Rhipicephalus sanguineus* (28.9%; 58/201), *Rhipicephalus turanicus* (19.9%; 40/201) and *Rhipicephalus haemaphysaloides* (14.9%; 30/201). Among *Hyalomma* ticks, the most abundant species was *Hyalomma anatolicum* (42.3%; 85/180) followed by *Hyalomma dromedarii* (17.8%; 32/180), *Hyalomma scupense* (15.6%; 28/180), *Hyalomma kumari* (10.6%; 19/180) and *Hyalomma isaaci* (8.9%; 16/180). The genus *Ixodes* was represented by only one species, *Ixodes kashmiricus* (100%; 32/32). Of the total examined domestic hosts (264), the most abundant were goats (28%; 74/264) and sheep (27.3%; 72/264), followed by cows (18.6%; 49/264), dogs and horses (9.5%; 25/264 each), and camels (7.2%; 19/264). With an overall prevalence of infestation (48.5%; 129/266), the highest prevalence was found on goats (56.8%; 42/74) followed by sheep (51.4%; 37/72), dogs (48%; 12/25), cows (42.9%; 21/49), camels (38.1%; 8/19) and horses (36%; 9/25). With an overall tick burden of 2.8 ticks per examined host, the highest tick burden was noted on goats (3.6; 268/74), followed by sheep (3.2; 232/72), cows (2.6; 129/49), dogs (1.7; 43/25), camels (1.5; 28/19), and horses (1.1; 29/25). The details about the life stage, associated hosts, and collection site of each tick species are given in Table 2.

**Table 2:** Information related to tick species, their hosts and collection sites, as well as PCR results of associated *Borrelia* spp.

Tick species	Tick life stages			Total Ticks	Hosts species (No. of infested/No. of examined)	Tick collection sites	PCR details		
	Female	Male	Nymph				No. of ticks subjected to PCR	No. of infected ticks	*Borrelia* spp. (amplified partial fragment)
*I. kashmirensis*	19	6	7	32	Goats (3/3), Sheep (2/4)	Shangla	7F, 3 N	–	–
*Ha. bispinosa*	30	22	10	62	Goats (3/6), Sheep (5/7)	Bajaur, Dir Upper, Dir Lower, Malakand, Mohmand, Shangla	7F, 3 N	–	–
*Ha. cornupunctata*	51	26	17	94	Goats* (8/10), Sheep (4/6)	Bajaur, Dir Upper*, Dir Lower*, Malakand, Mansehra*, Mohmand, Shangla	7F, 3 N	4	*Borrelia* sp. (*flaB*)
*Ha. danieli*	13	5	3	21	Goats (5/8)	Dir Upper	7F, 3 N	–	–
*Ha. kashmirensis*	34	24	8	66	Goats (7/9), Sheep (6/8)	Bajaur, Dir Upper, Dir Lower, Malakand, Mohmand, Shangla	7F, 3 N	–	–
*Ha. montgomeryi*	25	16	4	45	Goats (4/6), Sheep (5/5)	Bajaur, Dir Upper, Dir Lower, Malakand, Mohmand, Shangla	7F, 3 N	–	–
*Ha. sulcata*	17	8	3	28	Goats* (3/5), Sheep (4/5)	Bajaur*, Dir Upper, Dir Lower, Malakand, Mohmand, Shangla	7F, 3 N	2	*Borrelia* sp. (*flaB*)
*Hy. anatolicum*	46	21	18	85	Camels (2/6), Cows (4/6), Dogs (1/4), Goats (1/3), Horses (2/7), Sheep (2/5)	Bajaur, Malakand, Mohmand, Mardan, Swabi	7F, 3 N	–	–
*Hy. dromedarii*	14	8	10	32	Camels (3/3), Cows (1/4), Sheep (1/6), Horses (1/5)	Charsadda, Malakand, Mardan, Mohmand, Nowshera, Peshawar, Swabi	7F, 3 N	–	–
*Hy. kumari*	9	7	3	19	Goats (3/7), Sheep (2/8)	Mohmand, Nowshera	7F, 3 N	–	–
*Hy. scupense*	9	11	8	28	Cows (4/10), Horses (4/9)	Charsadda, Malakand, Mardan, Mohmand, Nowshera, Peshawar, Swabi	7F, 3 N	–	–
*Hy. isaaci*	6	5	5	16	Cows (2/7), Goats (1/5), Sheep (1/5)	Charsadda, Mardan, Mohmand, Nowshera, Peshawar, Swabi	7F, 3 N	–	–
*R. haemaphysaloides*	12	8	10	30	Camel (2/8), Cows (2/6), Dogs (2/5), Goats (1/2), Sheep (1/3), Horse (2/5)	Bajaur, Charsadda, Mardan, Malakand, Mohmand, Nowshera, Peshawar	7F, 3 N	–	–
*R. microplus*	29	21	23	73	Camel (1/4), Cows* (6/8), Dogs (1/4), Goats (1/3), Sheep* (2/4)	Bajaur, Charsadda*, Dir Lower, Malakand, Mardan, Swabi	7F, 3 N	3	*B. theileri* (*flaB*)
*R. sanguineus*	24	19	15	58	Dogs (6/80), Cows (1/3), Goats (1/4), Sheep (1/3)	Bajaur, Dir Lower, Malakand, Mardan, Peshawar, Shangla, Swabi	7F, 3 N	–	–
*R. turanicus*	17	13	10	40	Cows (1/5), Dogs (2/4), Goats* (1/3), Sheep (1/3)	Bajaur, Malakand*, Mohmand, Shangla, Swabi^*^	7F, 3 N	2	*B. theileri* (16S rRNA and *flaB*)
	359	220	150	729	–	–	160	11	–

*Associated with *Borrelia* spp. positive ticks.

### Sequences analyses

The partial fragments of borrelial *flaB* were amplified in 11 ticks, while partial fragments of borrelial 16S were amplified in 2 *flaB*-positive ticks. The attempts to amplify 16S rRNA in the remaining 9 *flaB*-positive ticks were unsuccessful. Overall, 4 sequences (1 forward and 1 reverse per positive tick sample) were obtained for 16S rRNA, while 22 longer sequences (1 forward and 1 reverse per positive tick sample) were obtained for *flaB.* A subset of sequences of 16S rRNA obtained from the genomic DNA of *R. turanicus* from goats and cattle were found to be identical, which resulted in consensus sequences of 644 bp. Similarly, all long sequences of *flaB* within each subset were identical in the following ways: (a) sequences obtained from *R. microplus* from sheep and cattle were identical, (b) sequences obtained from *R. turanicus* from goats were identical, (c) sequences obtained from *Ha. cornupunctata* from goats were identical, and sequences obtained from *Ha. sulcata* from goats were identical. These four subsets resulted in consensus sequences of 587, 522, 547, and 559 bp, respectively. Besides the long sequences for *flaB*, 11 short sequences (1 forward per positive tick sample) were obtained from their corresponding positive PCR samples.

### Detection of *Borrelia* spp. in ticks

The consensus sequences of borrelial 16S rRNA obtained from genomic DNA of *R. turanicus* from goats shared a maximum identity of 100% with *B. theileri*. The long consensus sequences of borrelial *flaB* obtained from the same samples also showed a maximum identity of 99.40% with *B. theileri*. Other long consensus sequences of borrelial *flaB* obtained from tick’s genomic DNA showed their BLAST identities in the following ways: (a) *Borrelia* sp. detected in *R. microplus* from cows and sheep showed a maximum identity of 100% with *B. theileri*, (b) *Borrelia* sp. detected in *Ha. cornupunctata* from goats depicted a maximum identity of 99.32% with a *Borrelia* sp., and (c) *Borrelia* sp. detected in *Ha. sulcata* from goats displayed a maximum identity of 99.75% with *Borrelia* sp. Among the determined RF borreliae, all these sequences were found close to *B. theileri* followed by *B. lonestari*.

Considering 16S rRNA-based detection of *Borrelia* spp., only *R. turanicus* was positive, resulting in an overall infection rate of 0.3% (2/160). When considering *flaB*-based detection of *Borrelia* spp., four tick species, including *R. microplus*, *R. turanicus*, *Ha. sulcata*, and *Ha. cornupunctata* were positive, yielding an overall infection rate of 6.9% (11/160). Notably, within the overlapped region, the short and long sequences of *flaB* were identical, confirming the BLAST identities of each other. However, only long sequences were used in phylogenetic analyses. Table 2 gives details about *Borrelia* spp., including the associated hosts and the corresponding geographical sites.

The obtained sequences were submitted to GenBank under the following accession numbers: 16S rRNA OR561043 (*B. theileri* haplotype detected in *R. turanicus* from goats); and *flaB* OR574987 (*B. theileri* haplotype detected in *R. turanicus* from goats), OR574986 (*B. theileri* detected in *R. microplus* from cows and sheep), OR574984 (*Borrelia* sp. detected in *Ha. cornupunctata* from goats), OR574985 (*Borrelia* sp. detected in *Ha. sulcata* from goats).

### Phylogenetic analysis

**Figure 2:**
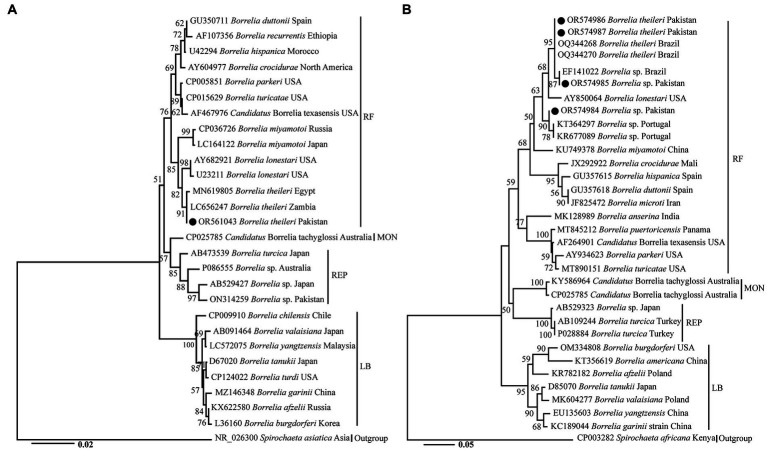
The phylogenetic trees were constructed for *Borrelia* spp. based on 16S rRNA **(A)** and *flaB* **(B)**, with *Spirochaeta asiatica*, and *Spirochaeta africana* serving as the outgroups, respectively. The Neighbor-Joining method was utilized with 1,000 replicates for bootstrap analysis. GenBank accession numbers, species names, and geographic locations were assigned to all sequences. *Borrelia* spp. detected in this study are indicated by a black circle. RF, MON, REP, and LB represent relapsing fever, monotreme-associated, reptile-associated, and Lyme *Borrelia*e, respectively.

A phylogenetic tree was obtained based on 16S rRNA, in which *B. theileri* detected in *R. turanicus* from goats in the present study clustered with the same species from Egypt and Zambia (Figure 2A). Furthermore, this species appeared in a monophyletic group alongside *B. lonestari* and *B. miyamotoi*. Another phylogenetic tree was obtained based on *flaB* (Figure 2B), revealing the clustering of *Borrelia* spp. detected in the current study in the following manner. The haplotype of *B. theileri* found in *R. microplus* from sheep and cows, as well as another haplotype of *B. theileri* found in *R. turanicus* from goats clustered with the corresponding species from Brazil. The *Borrelia* sp. found in *Ha. sulcata* from goats clustered with undetermined *Borrelia* sp. from Brazil, while the *Borrelia* sp. found in *Ha. cornupunctata* from goats clustered with undetermined species from Portugal. Additionally, these species also formed a monophyletic group along with *B. lonestari* and *B. miyamotoi*.

## Discussion

Compared to LB borreliae, RF borreliae have received less attention globally (26). Similarly, despite reported RF cases (58), the association of borreliae with ticks in in Asia in general and in Pakistan in particular is poorly known. Neglecting RF borreliae can have significant adverse consequences for both public and animal health. To address this knowledge gap, we conducted *Borrelia* spp. detection in 16/160 hard ticks collected from six different domestic animals across different geographical areas. In addition to detecting *B. theileri* in *R. microplus*, our study represents, to the best of our knowledge, the first report of *B. theileri* in *R. turanicus*, as well as the detection of *Borrelia* spp. in *Ha*. *cornupunctata* and *Ha*. *sulcata*.

In Pakistan, previous studies suggest that *Haemaphysalis* spp., *Hyalomma* spp., and *Rhipicephalus* spp. are commonly associated with domestic animals (38, 40, 42, 45, 59–63). Considering their distribution in previous studies, it can be inferred that *Haemaphysalis* spp. are prevalent in humid and vegetated areas, *Hyalomma* species prevalent in dry and desert areas, and *Rhipicephalus* spp. are abundant in humid and warm areas. Given that the current study area primarily consists of rangeland, cropland, and forested land surfaces, *Haemaphysalis* spp. and *Rhipicephalus* spp. were found the most abundant ticks in this study, in contrast to *Hyalomma* spp. Moreover, the higher abundance of *Haemaphysalis* and *Rhipicephalus* ticks could also be associated with their broader host range and greater number of main hosts.

Regarding HTRF borreliae, it is well studied that *B. theileri* is associated with the genus *Rhipicephalus*, especially *R. microplus* (26, 31). Along with *R. microplus*, the current study also detected this pathogen in *R. turanicus*, marking the earliest such finding. In contrast, the association of HTRF borreliae with the genus *Haemaphysalis* is poorly understood. This study and previous related reports (64, 65) suggest that there could be a considerable association between HTRF borreliae and *Haemaphysalis* ticks, which needs further investigation. Furthermore, apart from being found in hard ticks, previous studies have also detected *B. theileri* and other closely related undetermined *Borrelia* spp. in vertebrate hosts, including cattle, goats, and sheep (6, 31, 36). Given the known pathogenicity of *B. theileri* in domestic animals (26, 36, 37, 66), hard ticks could pose threats to domestic animals in the region. Several other factors in the studied region, including a high tick abundance, a large population of cattle and small ruminants, and their combined farming practices and unmonitored movements, could further exacerbate health threats.

Different analysis based on molecular data are considered powerful tools for the identification of biological species (67, 68). Furthermore, unlike the traditional systematic and taxonomy of TBRF borreliae, which was based on co-speciation of ticks and borreliae (26, 69), the advanced approach relies on molecular evidence (10, 26, 67–71). It is also studied that different molecular markers, including 16S rRNA and *flaB* are compatible in the case of identification and phylogenetic analysis of TBRF (67, 72). Therefore, 16S rRNA and *flaB* based molecular data was obtained for *Borrelia* spp. in the current study, which was subsequently subjected to phylogenetic analysis. The genetic variations in the detected *Borrelia* spp. could be associated with the difference in their tick hosts in general. Despite their mutual genetic variations, in the BLAST and phylogenetic analysis, these *Borrelia* spp. exhibited proximity to *B. theileri*, *B*. *lonestari* and *B. miyamotoi*. The observed closeness could be attributed to their shared niche, while most RF borreliae are associated with soft ticks, these are associated with hard ticks. *Candidatus* Borrelia texasensis, although considered to be transmitted by hard ticks (26, 73), did not cluster in the mentioned group.

## Conclusion

This study not only confirmed the presence of *B. theileri* in *R. microplus* but also provided the first documented evidence of *B. theileri* in *R. turanicus* collected on cattle and sheep, along with the detection of RF *Borrelia* spp. in *Ha. cornupunctata* and *Ha. sulcata* collected on goats. The study contributes to the expansion of the geographical and tick host range of RF borreliae, which, in turn, could support future research efforts focusing on veterinary health. Further studies should be encouraged to investigate the ticks-borne bovine borreliosis in order to reduce potential risks in the region.

## Data availability statement

The original contributions presented in the study are publicly available. This data can be found at: https://www.ncbi.nlm.nih.gov/; OR561043, OR574984-OR574987.

## Ethics statement

The animal studies were approved by Ethical permission for conducting this study was granted under the reference number Dir/A&R/AWKUM/2018/1410 by the Advanced Studies and Research Board of Abdul Wali Khan University, Mardan (AWKUM). Oral permissions were obtained from the owners of the animals, and the animals were treated with care and handled gently during the tick collection process. The studies were conducted in accordance with the local legislation and institutional requirements. Written informed consent was obtained from the owners for the participation of their animals in this study.

## Author contributions

MK: Data curation, Formal analysis, Investigation, Methodology, Software, Validation, Visualization, Writing – original draft, Writing – review & editing. MA: Data curation, Funding acquisition, Investigation, Methodology, Project administration, Resources, Software, Writing – original draft, Writing – review & editing. ADA: Formal analysis, Funding acquisition, Investigation, Methodology, Project administration, Resources, Writing – original draft, Writing – review & editing. TT: Data curation, Formal analysis, Investigation, Software, Writing – review & editing. S-CC: Data curation, Formal analysis, Funding acquisition, Investigation, Methodology, Resources, Validation, Visualization, Writing – original draft, Writing – review & editing. C-CC: Data curation, Funding acquisition, Investigation, Methodology, Project administration, Software, Writing – original draft, Writing – review & editing. AIA: Conceptualization, Data curation, Funding acquisition, Investigation, Methodology, Project administration, Resources, Supervision, Writing – original draft, Writing – review & editing.
